# Effects of Local Ischemic Compression on Upper Limb Latent Myofascial Trigger Points: A Study of Subjective Pain and Linear Motor Performance

**DOI:** 10.1155/2019/5360924

**Published:** 2019-03-04

**Authors:** Danilo Esparza, Arian R. Aladro-Gonzalvo, Yves Rybarczyk

**Affiliations:** ^1^School of Physiotherapy, Universidad de Las Américas, Quito, Ecuador; ^2^Intelligent & Interactive Systems Lab (SI^2^ Lab), School of Engineering in Computer Systems, Universidad de Las Américas, Quito, Ecuador; ^3^Faculty of Nursing, Pontifical Catholic University of Ecuador, Quito, Ecuador

## Abstract

**Objective:**

To analyse the effect of the manual ischemic compression (IC) on the upper limb motor performance (MP) in patients with LTrPs.

**Materials and Methods:**

A quasiexperimental study was performed in twenty subjects allocated to either patients group with LTrPs (PG, n=10) or healthy group with no symptoms (HG, n=10). Subjective pain and linear MP (movement time and Fitts' Law) were assessed before and after a linear tapping task. Data were analysed with mixed factorial ANOVA for intergroup linear motor performance differences and dependent t-student test for intragroup pain differences.

**Results:**

PG had a linear MP lower than the HG before treatment (*p* < 0.05). After IC, the PG showed a significant decrease of pain (4.07 ± 1.91* p* < 0.001). Furthermore, the movement time (15.70 ± 2.05* p* < 0.001) and the Fitts' Law coefficient (0.80 ± 0.53* p* < 0.001) were significantly reduced. However, one IC session did not allow the PG to get the same MP than the HG (*p* < 0.05).

**Conclusion:**

The results suggest the IC effectiveness on pain and MP impairment in subjects with LTrPs. However, the MP of these patients is only partially improved after the IC application.

## 1. Introduction

Myofascial syndrome is a clinical condition characterized by muscle pain related to Myofascial Trigger Points (MTrPs) [1]. The MTrPs are usually associated with (i) hyperalgesia, (ii) referred pain, (iii) behavioural perturbations, and (iv) functional limitations [2]. A MTrP is defined as a hypersensitive nodule located in skeletal muscles [3]. Two kinds of MTrPs are clinically described: latent and active. Latent Trigger Points (LTrPs) can be developed by maintaining a shortened muscle activation for a long period of time, exaggerated muscle contraction, or repeated physical activity. LTrPs pain can be triggered by digital compression, stretch, or overload [1]. On the contrary, Active Trigger Points (ATrPs) are (i) spontaneously triggered, (ii) may cause chronic and referred pain, and (iii) are associated with muscle weakness, reduced range of motion, and paresthesias [4–6]. Fear of pain, pain itself, and muscle injury [7] can cause muscle dysfunctions in both ATrPs and LTrPs.

Several studies report alterations in muscle function of subjects with LTrPs, such as decreased shoulder muscle strength in both dominant and nondominant sides [8]; EMG activity increased in the antagonist and synergists muscles [9, 10]; muscle fatigue increased and overload of motor units close to the LTrPs [11]; and muscle activity pattern modification in the upper limb kinematic chain [12]. Moreover, LTrPs contribute to the development of muscle cramps, restricted joint range of motion, muscle weakness, and accelerated fatigability [13]. However, to our knowledge none of the previous works were interested in studying the upper limb pain and its consequence on the motor performance (MP) in patients with LTrPs. These aspects are relevant, because a deficiency in the proximal segment of the upper limb kinematic chain may generate alterations in the MP of the distal segment and its functionality [14].

Common tasks to analyse the upper limbs MP is the finger tapping task [15]. This task allows spatiotemporal parameters modification of movement and motor control analysis [16]. The finger tapping task consists of reaching two distant targets as fast and accurate as possible. It is frequently used to assess MP in the motor sequences learning process (i.e., the motor system efficiency) or in patients with neurological disabilities [17–19]. In addition, this task permitted MP analysis according to the Fitts' Law [20]. This law provides a mathematical motor control model, which predicts the movement time (MT) according to a certain index of difficulty (distance between targets and width of the targets).

Moreover, different manual therapy techniques are applied to subjects with MTrPs, in order to evaluate its effect on pain and functionality [21]. Results show muscle pain decrease and joint mobility increase [22–24]. An efficient technique for treating MTrPs and reducing muscle pain is manual local ischemic compression (IC) [25, 26]. On the basis of these developments and considerations, if a muscle alteration is the cause of motion perturbation in the proximal kinematic chain, we make the assumption that (i) pain would influence arm's MP in subjects with LTrPs and (ii) a possible pain reduction by IC treatment would improve the kinematic performance. These questions are addressed through a subjective pain and linear MP (MT and Fitts' Law) analysis in the upper limb, before and after IC application on patients with LTrPs.

## 2. Methods

### 2.1. Design

A quasiexperimental study was performed in twenty subjects, aged between 20 and 47 years old (mean age = 28; SD = 7.4). The subjects were allocated in a healthy group (HG) (n=10) or a patient group (PG) (n=10), after a clinical assessment performed by an experienced physical therapist.

### 2.2. Participants

Subjects were invited to the experiment via email and by ads posted in the University. Before enrolment, all subjects signed an informed consent form to participate in the study, which complied with the ethical criteria established in the Helsinki Declaration (as revised in 2013) on research projects and was approved by the ethics committee of the Caen University.

To be included in the PG, the individuals had to show: (i) a palpable taut band in at least two skeletal muscles, (ii) a hypersensitive tender spot, (iii) referred pain of the MTrP in response to compression, (iv) jump signs like (winces cry and/or withdraw), and (v) a local twitch response caused by the snapping palpation of the taut. LTrPs were considered positive if two or more criteria were identified [27]. The individuals that (i) received a pharmacological treatment, (ii) followed a physical rehabilitation program, (iii) had a neck or shoulder surgery in the previous year, or (iv) suffered severe inflammatory, neurologic, or traumatic disturbances were excluded from the study. The HG participants neither had LTrPS nor received any treatment.

### 2.3. Tapping Task

An adapted version of the “Plate Tapping Test”, from the Eurofit Test Battery, was used to evaluate the upper limbs linear MP. The subjects were sitting at a comfortable distance from the table with their dominant hand setting on the border of the table in cubital deviation, such as the ring finger, the middle finger, and the index finger perfectly leaned on the target. The distance between the subject and the border of the table was adjusted according to the arm's length of each individual. Thus, the middle finger of the dominant hand leaned on the central line, which was aligned with the sternum. The participant's task was to stretch their dominant arm, without moving their trunk, in order to reach each target as fast as possible. The patients received instruction not to rotate the trunk maintaining the shoulders in contact with the backrest. Each participant executed two trials of twenty-five movements (50 tapping trials in total), after hearing the auditive signal “Go”. Trials were separated by 2 min interval. The average time of the twenty-five movements was used for the statistical analysis.

### 2.4. Measurement of Pain Intensity by Visual Analog Scale (VAS)

Subjects had to express pain level by manipulating a slider on a scale. The VAS is a subjective measurement, which is used to quantify pain intensity felt by the individuals. This is a graduated scale used to gauge pain, from no pain (0) to maximum pain (10). The subject's task was to move a slider over a 0 to 10 cm graduated ruler. The reliability of the VAS was demonstrated by Jensen et al. [28].

### 2.5. Identification of the MTrPs

A physiotherapist expert in the MTrPs exploration performed an upper dominant limb physical examination. Four LTrPs were identified and evaluated on the following muscles: (i) middle trapezius, (ii) levator scapulae, (iii) infraspinous, and (iv) teres minor. These muscles were chosen because of their involvement in shoulder movements and LTrPs prevalence in healthy subjects [29].

Subjects were prone lying in a comfortable position, arms in abduction, and elbows in flexion and leaned on the table. This position ensured muscle fiber relaxation, facilitating MTrPs localization and evaluation [12]. The points were manually identified, as suggested by the literature through a manual pressure of approximately 2 to 4 kg/cm^2^ and a velocity pressure of 1 kg/cm^2^/s [30, 31]. The amount of pressure and velocity were applied according to the evaluator's clinical experience. The exploration of two consecutive points was separated by a 20 s interval [30, 31]. All LTrPs evaluation was performed by the same physiotherapist, in order to avoid an interpersonal variability in the identification and evaluation.

### 2.6. Local Ischemic Compression (IC)

In the same position as described in the MTrPs identification, the physiotherapist applied a manual pressure using his thumb on the LTrPs until reaching the PG subject's pain threshold. This threshold was identified by either an oral expression or a visible muscle contraction. Then the pressure had to be maintained during 30 s. If the patient expressed pain reduction before the 30 s, the pressure was gradually increased until reaching a new subjective pain threshold self-reported. If a patient experienced more pain during treatment, then pressure was decreased until attaining tolerable level. This IC technique was preferred because it reduces pain more efficiently than other manual therapies [22, 32]. For example, a significant pain intensity decrease was reported when a short duration and a high intensity IC were performed [33]. In order to dissipate the discomfort post IC, effleurage was applied during 10 s after treatment.

### 2.7. Fitts' Law

Fitts' Law is a mathematical relationship that predicts the MT according to an index of difficulty (ID). Several studies have demonstrated that this equation is a universal empirical model for characterizing the human MP. Here, it is used as an advanced analysis to gauge the MTrPs impact on the correlation between speed and accuracy of the movement. The general formulation of the law is as follows:

(1) $$\begin{array}{c} MT=a.ID+b \end{array}$$

where a and b are constants that depend on the characteristic of the effector and are determined empirically by linear regression.

In ideal conditions, b is equal to zero and (1) can be simplified as

(2) $$\begin{array}{c} MT=a.ID \end{array}$$

The ID is based on two factors: the movement distance (A) and the target width (W), such as

(3) $$\begin{array}{c} ID={log}^{2}\left(\;\frac{A}{W}+1\;\right) \end{array}$$

In other words, the law claims that the larger the movement distance is and the smaller the target is, the longer MT will take. In the experiment, the distance between the two targets is 60 cm and the target width is 4.5 cm. Therefore, the value of the ID is

(4) $$\begin{array}{c} ID={log}^{2}\left(\;\frac{60}{4.5}+1\;\right)=3.84 \end{array}$$

Thus, the correlation referential values (aREF) between speed and accuracy in the pre and posttest are, respectively,

(5) aREFpre=MTHGpreID

(6) aREFpost=MTHGpostID

MT HGpre and MT HGpost are the average MT of the HG before and after treatment, respectively. In order to analyse the MT of patients with LTrPs regarding Fitts' Law, the difference between the referential coefficient (aREF) and each individual coefficient of the HG (ai) was compared to the difference between the referential coefficient (aREF) and each individual coefficient of the PG (ai), such as

(7) $$\begin{array}{c} \Delta i=\left|\;ai-aREF\;\right| \end{array}$$

### 2.8. Intervention Protocol

The participants were asked about discomfort and pain perception in the upper limb. Before starting the experiment, all subjects were submitted to an MTrPs clinical examination and were allocated to PG or HG according to the presence of LTrPs. In order not to revive pain, the clinical examination was carefully performed. Then the first stage of the experiment consisted of the tapping task completion in both groups. Afterwards, the muscle pain perception in the PG was measured by VAS. Finally, the treatment with IC was applied on PG. This procedure was repeated 48 hours after treatment, without the IC application. This 48 hours' delay was determined considering that pain may persist 24 hours after the treatment [34].

### 2.9. Statistical Methods

The statistical analysis was performed with SPSS, version 22.0. Values are presented as mean (M) and standard deviation (± SD). MP mean was calculated by averaging measures taken during the two repeated trials. Two separate mixed ANOVA were completed (2 Groups x 2 Measures) to compare mean differences between groups for the MT and the Fitts' Law analysis of the tapping test. Furthermore, a paired t-student analysis was performed in order to compare mean differences within PG for the subjective pain perception. A value of* p* < .05 was considered statistically significant.

## 3. Results

**Figure 1:**
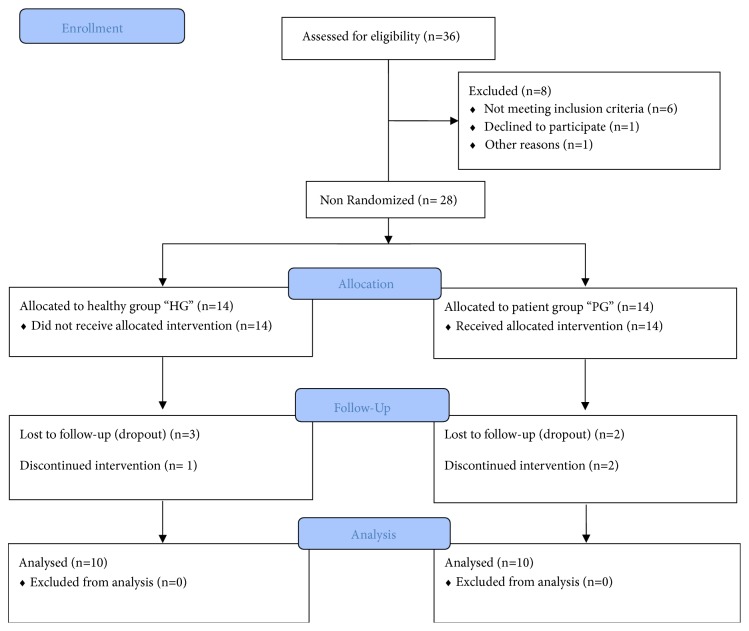
Participants flow diagram.

From the twenty-eight subjects who met with the inclusion criteria and who voluntarily accepted to participate in the study, only twenty achieved the study. The losses of observation were caused by the fact that (i) three HG participants and two PG participants dropped out the study; (ii) the data of two subjects of the PG were lost due to error recording during the first evaluation; and (iii) one participant of the HG was excluded in order to have the same number of participants in each group (Figure 1).

### 3.1. Pain

**Figure 2:**
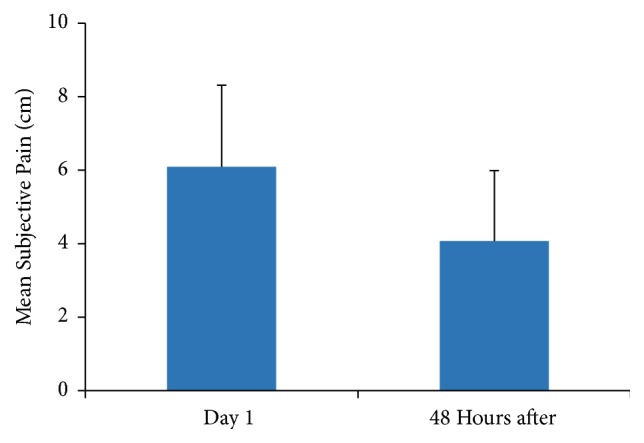
Mean and standard deviation differences in the PG's subjective pain perception before and after the treatment (*p* < 0.05).

The paired t-student analysis for the pain subjective perception shows a significant difference before and after IC in the PG (*p *= 0.001; 95% IC: 1.08 to 2.95) which suggests that the modification in the pain perception can be attributed to the IC treatment (Figure 2).

### 3.2. Motor Performance

**Figure 3:**
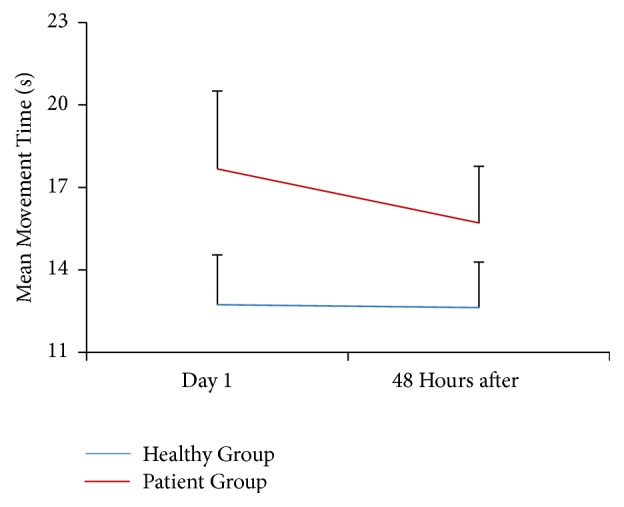
Mean and standard deviation movement time for the tapping task in the HG and the PG. Intra- and intergroup differences between Day 1 and 48 hours after treatment (*p* < 0.05). Lines represent the means for each group.

The ANOVA analysis comparing the MT shows an interaction group-measure (*p* = 0.004). The post hoc analysis shows a significant difference in the time required to complete the tapping test between the PG (17.67 ± 2.83s) and the HG (12.73±1.81 s) before IC (*p* = 0.001; 95% IC: 2.71 to 7.18) and after IC (PG=15.70 ± 2.05 s; HG=12.62 ± 1.65 s) (*p* = 0.002; 95% IC: 1.33 to 4.84) (Figure 2). In addition, there was a difference in the MT average before and after IC in the PG (*p* = 0.001; 95% IC: -2.81 to -1.12), whereas no difference was reported in the HG (*p* = 0.795; 95% IC: -0.95 to 0.74) (Figure 2). This result means that the PG subjects are slower than the HG subjects at the first stage of the experiment (Day 1), but they significantly improve their performance after the IC treatment (48 hours later). However, the completion time required by the PG continues to be higher than the HG. This last group keeps its performance at the same level across the experiment (Figure 3).

**Figure 4:**
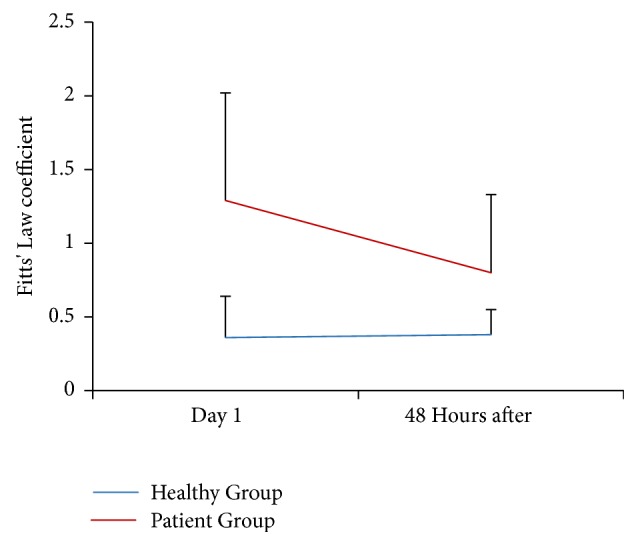
Interaction between the referential coefficient and the coefficient of each individual during the tapping task. Intra- and intergroup differences between Day 1 and 48 hours after treatment (*p* < 0.05). Lines represent the means for each group.

The ANOVA for the Fitts' Law analysis shows a significant interaction (Figure 4). The difference was observed between the PG and the HG before (mean difference = 0.93; 95% IC: 0.40 to 1.45,* p* = 0.002) and after treatment (mean difference = 0.43; 95% IC: 0.53 to 0.80, p = 0.03). It means that the correlation between speed and accuracy in patients with LTrPs is different than the one predicted by Fitts' Law. This difference persists after treatment but tends to be reduced. The post hoc intragroup comparison before versus after treatment shows no difference in the HG (mean difference = -0.16; 95% IC: -0.23 to 0.20,* p* = 0.88). On the contrary, the same test performed on the PG shows a significant difference (mean difference =0.49; 95% IC: 0.27 to 0.70,* p* = 0.001) (Figure 4). This result suggests that the treatment has a relevant effect, in the way that the patients' MP is closer to the model predicted by the Fitts' Law.

## 4. Discussion

The objective of this study was to analyse the effect of the musculoskeletal pain on MP in subjects with LTrPs. The results show that (i) pain resulting from LTrPs causes an alteration in the execution of the tapping task, and (ii) the IC treatment reduces pain and partially improves the upper limbs MP. The limitations of the study were that the therapist who administered both the intervention protocols and the evaluation could not be blinded. Also, the experimental conditions did not permit to test Fitts' Law with different indexes of complexity.

The IC application significantly changed the values of the subjective pain perception registered in patients with LTrPs. The subjective pain was reduced 2.02 cm in the 48 hours that followed the treatment by IC. On one hand, this result is consistent with previous studies that demonstrated a significant pain reduction in patients with MTrPs and, consequently, the efficiency of the IC technique [22, 26, 33]. On the other hand, the outcomes of the present study support other researches that tend to demonstrate that the manual therapy is not enough to fully improve the muscle contraction (i.e., muscle activation and strength production) [35] and the motor control. Two studies suggest that pain perception drop does not necessarily translate into a significant functional improvement in joint amplitude and quality of life [36, 37].

The mechanism that permits the IC to reduce pain is based on a pressure that locally dilates the sarcomeres [6]. This dilatation increases the blood flow, which allows a drainage of the cellular metabolic subproducts commonly associated with the pain production in the MTrP. Thus, this technique aids to restore a normal metabolic functioning of the affected tissues [38]. The thalamus and the limbic system are critically involved in the subjective pain perception. Since the nociceptive inputs are subthreshold, no action potential is produced in the CNS and, consequently, the subject does not perceive pain [39]. According to Mense two aspects may explain the mechanism of the LTrPs [40]. The first one is related to subthreshold nociceptive signals sent to the spinal cord dorsal horn. This phenomenon should stimulate the CNS without producing pain perception, which is the characteristic of the LTrPs. The second aspect is the existence of inefficient synapses in the dorsal horn of the spinal cord. Finally, it was proposed that MTrP compression alters the activity of the autonomic nervous system via the prefrontal cortex to reduce subjective pain [41].

The study shows a MP alteration for executing tapping task in subjects with LTrPs. The average time to execute the movement was 4.94 s slower for the PG before treatment than for the HG. After the IC treatment, the difference was reduced to 3.08 s in average. The MT for the HG was maintained around the same value in the two sessions of evaluation, whereas this time significantly reduced in the PG after treatment (1.97 s). A study in patients with LTrPs did not find significant differences in arm movement measuring reaction time between LTrPs and control group [42]. This suggests that LTrPs do not alter the muscle response for the onset of upper limbs movements. This observation could explain that the difference found in the MT in this study is not due to the relationship stimulus/reaction of the muscle contraction. Our result is rather explained by the presence of the accelerated fatigability observed in patients with LTrPs [13].

On the other hand, these results confirm the hypothesis that a significant pain reduction improves the MP in subjects with LTrPs. This study is the first to demonstrate that the manual pain reduction is not enough to enable the patients to reach a normal MP, taking into account the fact that the PG cannot get the same performance than the HG. Two possible reasons may explain such an outcome. First, the pain reduction by the IC application does not permit a normal recovery of the muscle recruitment pattern. This interpretation is supported by studies that show an alteration of the muscle activation pattern in LTrPs subjects [43, 44]. Probably, the MT alteration is related to the EMG activity increase in the antagonist [9] and synergist muscles [9, 10]. This phenomenon involves an asynchrony in the upper limb muscle activity, which would be the cause of a muscle fatigue and an overload of the motor units close to the LTrP [11]. In this case, the IC should be completed by another treatment specifically focused on the motor control improvement of the limbs affected by the LTrPs. For instance, the tapping skill relearning would be necessary, considering that the ability to target a specific component of movement requires greater skills and increased levels of attention and accuracy than the contraction of all muscles (e.g., strength training) [45]. Second, the subjective pain perception is not reliable enough to precisely determine the real level of pain [46]. In order to identify which of the two explanations is correct, a complementary study including a more challenging motor task and a more objective measurement of pain threshold should be carried out.

In terms of clinical application, these findings suggest that the manual pain reduction would be insufficient and that it would have to be completed by a reeducation of the muscle activation sequence in order to totally recover the MP (time and accuracy). Thus, a possible therapeutic program for patients with LTrPs would be as follows: (i) reduction of pain, because it can impede the cortical neuroplastic changes associated with a novel motor-skill acquisition [47], (ii) inhibition of the activity of the synergist and antagonist muscles and reduction of the overloading of the motor units close to LTrPs, by a technique of proprioceptive neuromuscular facilitation [48, 49], and (iii) improvement of the muscle activation sequence through a novel motor-skill training, which is considered relevant for treating patients with musculoskeletal pain [50].

The tapping task enables us to study the MP in terms of Fitts' Law, which consists of evaluating the MT according to the amplitude of this movement and the size of the targets [20]. This law provides a mathematic model of the MT that is exclusively specified by the distance and the precision of the trajectory. We found that the LTrPs MP does not follow the Fitts' Law. However, such patients get closer to this law after IC treatment. It means that their motor control significantly improves without being exactly the same as in normal conditions. This result tends to explain the lower MP of the PG according to the hypothesis of a motor control deterioration. In this study, the Fitts' Law provides a more advanced analysis of the ipsilateral motor control in subjects with musculoskeletal disorders, considering that people with LTrPs do not present quantifiable clinical manifestations of MP deterioration. This is important from the clinical point of view, since the tapping task application could unmask MP deficits in people with LTrPs. The tapping task has been largely used to evaluate motor control and motor skill of the upper limbs. This task is frequently applied to provide a quantitative assessment of the MP in patients with Parkinson, ataxia, Alzheimer, Korsakoff syndrome, stroke, among others [51]. Virtually, no study made use of the tapping to evaluate the MP in patients with LTrPs, although this test is not difficult to apply and can be easily implemented in standard clinical practices.

## 5. Conclusions

This study constitutes the first comprehensive evaluation of the linear MP in patients with LTrPs. The main outcome is a demonstration of the limitation in the completion time to execute a linear movement in such patients. Other finding is the confirmation that the IC application is effective reducing pain. However, the short-term effect of the IC on pain perception is only partially reflected in terms of the MP recovery. Finally, this work suggests that an early detection of LTrPs could prevent the development of abnormal motor control, which may have consequences on the muscle activity and the emergence of ATrPs.

## Data Availability

The data used to support the findings of this study are available from the corresponding author upon request.
